# IgG-single-chain TRAIL fusion proteins for tumour therapy

**DOI:** 10.1038/s41598-018-24450-8

**Published:** 2018-05-17

**Authors:** Martin Siegemund, Felix Schneider, Meike Hutt, Oliver Seifert, Ines Müller, Dagmar Kulms, Klaus Pfizenmaier, Roland E. Kontermann

**Affiliations:** 1 Institute of Cell Biology and Immunology, University of Stuttgart, Allmandring 31, 70569 Stuttgart, Germany; 2 Department of Dermatology, Experimental Dermatology, TU-Dresden, 01307 Dresden, Germany; 3 Center for Regenerative Therapies TU Dresden, TU-Dresden, 01307 Dresden, Germany; 4 Stuttgart Research Center Systems Biology, University of Stuttgart, Nobelstraße 15, 70569 Stuttgart, Germany

## Abstract

Single-chain formats of TNF-related apoptosis inducing ligand (scTRAIL) can serve as effector components of tumour-associated antigen-targeted as well as non-targeted fusion proteins, being characterized by high tumour cell-specific induction of apoptosis through death receptor activation. We studied the suitability of immunoglobulin G as a scaffold for oligovalent and bispecific TRAIL fusion proteins. Thus, we developed novel targeted hexa- and dodecavalent IgG-scTRAIL molecules by fusing scTRAIL to the C-terminus of either light (LC-scTRAIL) or heavy immunoglobulin chain (HC-scTRAIL), or to both ends (LC/HC-scTRAIL) of the anti-EGFR IgG antibody hu225. The binding specificity to EGFR and death receptors was retained in all IgG-scTRAIL formats and translated into high antigen-specific bioactivity on EGFR-positive Colo205, HCT116 and WM1366 tumour cell lines, with or without sensitization to apoptosis by bortezomib. *In vivo*, therapeutic potential was assessed for one of the targeted variants, HC-scTRAIL, compared to the non-targeted Fc-scTRAIL. Both molecules showed a significant reduction of tumour volume and synergism with a Smac mimetic in a Colo205 xenograft tumour model. The IgG-scTRAIL format allows directing a defined, highly bioactive form of TRAIL to a wide variety of tumour antigens, enabling customized solutions for a patient-specific targeted cancer therapy with a reduced risk of side effects.

## Introduction

Therapeutic proteins based on the death ligand TNF-related apoptosis inducing ligand (TRAIL) have emerged as a novel therapeutic strategy in oncology^1^. TRAIL represents an excellent basis for anti-cancer biopharmaceutics due to its ability to induce apoptosis via death receptor activation preferentially in cancer cells in a p53 independent manner^2^. Of relevance, TRAIL has the capacity to target slow-growing cancer stem cells, too, which are considered as one major cause of tumour relapse^3^. However, constitutive or therapy-induced resistance to TRAIL-mediated apoptosis, e.g., by transcriptional down regulation of death receptors^4^ or upregulation of anti-apoptotic proteins like XIAP^5^ or Bcl-2^6^ has been described as well. In order to circumvent selection and expansion of TRAIL resistant tumour cells, one strategy is the generation of TRAIL variants or other TRAIL receptor agonists with enhanced activity, and/or the combination of such molecules with apoptosis sensitizers^1,7^. Insights into the principal mechanisms of receptor activation by ligands of the TNFR superfamily were instrumental for the development of second generation TRAILR agonists. In particular relevant for TRAIL receptors, the invention of stable, trivalent single-chain TNF family ligands^8–10^ and the knowledge of multivalent ligand-receptor interactions being required for apoptosis induction^11,12^ fostered the development of highly bioactive fusion protein formats for death ligands. Thus, single-chain TRAIL (scTRAIL) was subsequently used for generation of fusion proteins comprising dimerization domains. These constitute, with respect to TRAILR binding, hexavalent proteins. Such hexavalent TRAIL molecules fulfil the expected criteria of enhanced, tumour selective activity *in vitro* and *in vivo*^10,13–17^. The targeting of TRAIL to the cell surface of tumour or tumour stroma cells, as realized by antibody-mediated binding to specifically overexpressed antigens, e.g., growth factor receptors or cell adhesion molecules, has been shown to result in further enhanced bioactivity^1,18,19^.

Here, we report the generation and functional characterization of IgG-scTRAIL fusion proteins in different configurations, utilizing a humanized derivative of anti-EGFR IgG C225 (cetuximab) as a platform for oligomerization of TRAIL. High tumour-directed bioactivity combined with favourable pharmacokinetics suggests a potential use of IgG-scTRAIL in targeted therapy of cancer.

## Results

### Design and production of anti-EGFR IgG-single-chain TRAIL fusion proteins

**Figure 1:**
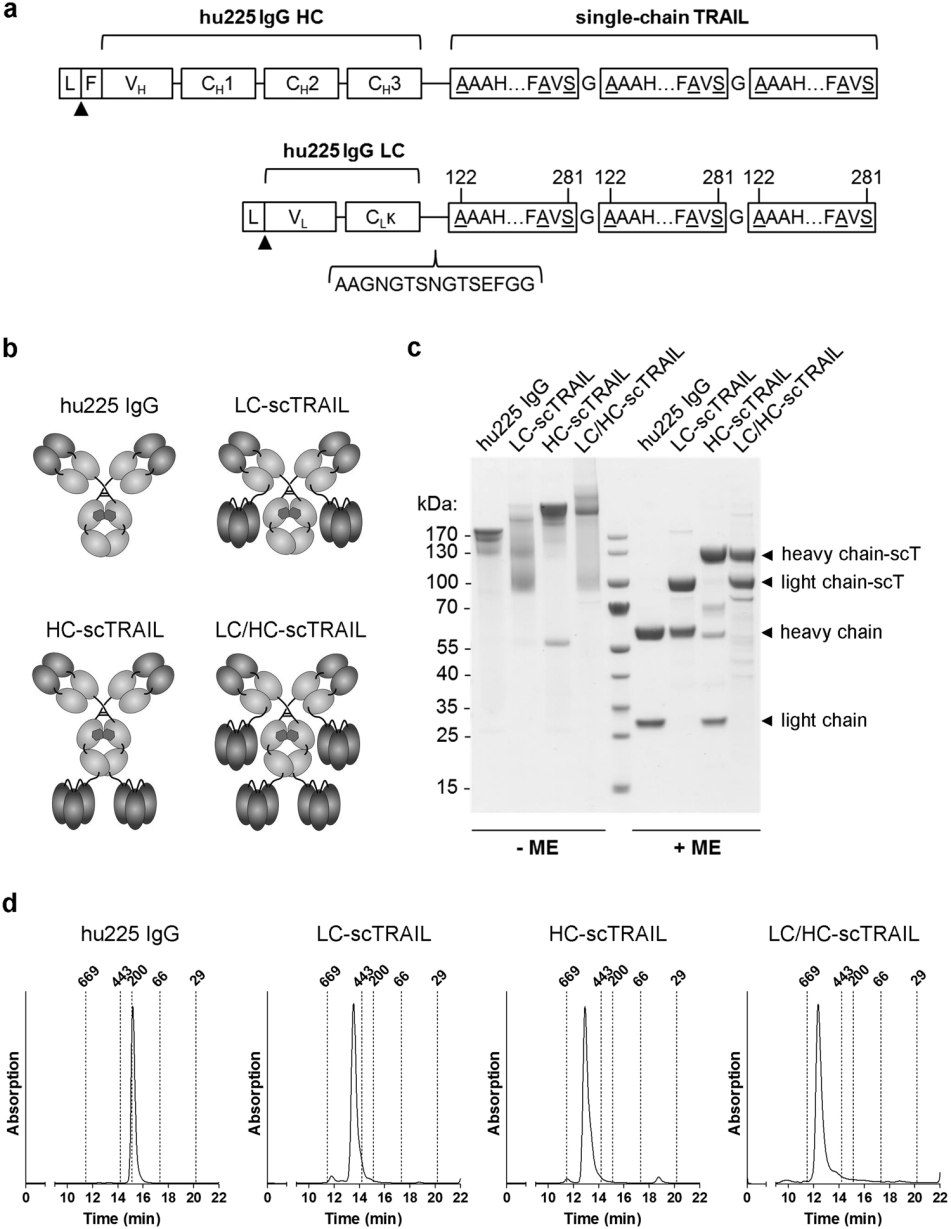
Structure and basic biochemical properties of IgG-scTRAIL fusion proteins. **(a**) Scheme of single-chain TRAIL fusion to the C-terminus of the anti-EGFR IgG1 heavy and light chain. Underlined aa residues at both termini of the TRAIL subunits indicate substitutions compared to wild-type TRAIL. C_H_2 and C_H_3 harbour mutations which abrogate ADCC/CDC functionality. A 15 aa residues peptide linker comprising a sequence motif with two N-glycosylation sites (AAGNGTSNGTSEFGG) was used for genetic fusion of C_H_3 or C_L_ domains with the first subunit of single-chain TRAIL. L, leader peptide; F, FLAG tag. (**b**) Drawings of full IgG-scTRAIL fusion proteins depicting control anti-EGFR huC225 IgG1 (hu225 IgG), fusion of scTRAIL to hu225 IgG light chains (LC-scTRAIL), fusion of scTRAIL to hu225 IgG heavy chains (HC-scTRAIL) and fusion of scTRAIL to both hu225 IgG chains (LC/HC-scTRAIL). **(c)** Hu225 IgG and scTRAIL fusion variants (5 µg each) were analysed by non-reducing (left) and reducing (right) SDS-PAGE and subsequent Coomassie staining. scT, single-chain TRAIL. (**d**) Hu225 IgG and scTRAIL fusion variants and were analysed by size exclusion chromatography under native buffer conditions. Elution times and molecular masses of globular reference proteins are indicated by dotted lines.

In this study, we used a humanized version (hu225) of EGFR-blocking antibody cetuximab as a basis to generate IgG-single-chain TRAIL (IgG-scTRAIL) fusion proteins. A scTRAIL derivative combining high bioactivity with enhanced thermostability (scTRAIL-FAVSGAA)^10^ was fused either to the C-terminal end of the light chain or the heavy chain comprising an effector-deficient Fc region. Moieties were joined by a peptide linker of 15 amino acid residues comprising two N-glycosylation sites (Fig. 1a). Three IgG-scTRAIL variants, characterized by hexavalent (LC-scTRAIL, HC-scTRAIL) or dodecavalent arrangement (LC/HC-scTRAIL) of scTRAIL, were generated (Fig. 1b). Upon transient expression and subsequent anti-FLAG affinity chromatography purification, all variants yielded soluble protein, composed of full-length polypeptide chains with the expected molecular masses and only low amounts of impurities or degradation products, as proven by reducing SDS-PAGE (Fig. 1c). Under non-reducing conditions, Fc-mediated dimerization of the molecules could be confirmed, although fusion of scTRAIL to the light chain resulted in a more diffuse pattern of bands. The large, dodecavalent variant LC/HC-scTRAIL (~375 kDa) produced lower yields than the hexavalent variants LC-scTRAIL and HC-scTRAIL (~262 kDa). Thus, yields of up to ∼9 mg purified protein per litre cell culture supernatant were obtained for HC-scTRAIL (Table 1). Importantly, all proteins eluted essentially as single peaks in size-exclusion chromatography (SEC), similar to the humanized IgG (hu225 IgG) produced under the same conditions (Fig. 1d).

**Table 1:** Biochemical properties of hu226 IgG and IgG-scTRAIL fusion proteins.

Molecule	M_r_ (kDa)	R_S_ (nm)	Yield^*^ (mg/L sup.)
hu225 IgG	148	5.0	11.2
LC-scTRAIL	261	6.5	6.7
HC-scTRAIL	261	7.1	9.4
LC/HC-scTRAIL	375	7.8	2.1

*Production in genetically engineered HEK293-S cells.

### EGFR-specific binding of IgG-scTRAIL fusion proteins

**Figure 2:**
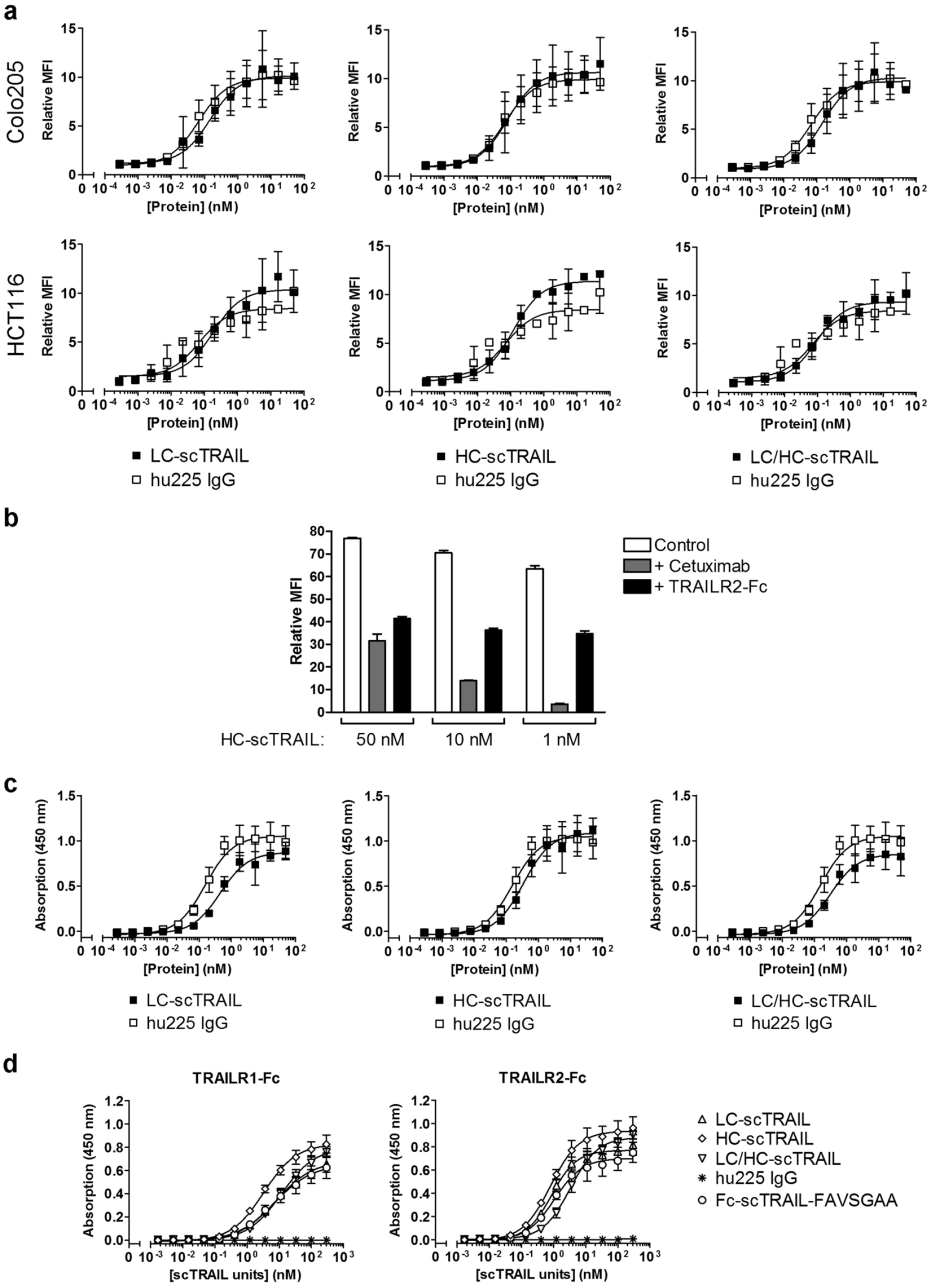
Binding of IgG-scTRAIL fusion proteins. (**a**) Combined binding of affinity-purified hu225 IgG-scTRAIL fusion protein variants to cell surface-expressed EGFR and TRAIL receptors was analysed on Colo205 and HCT116 cells, together with EGFR binding of sole hu225 IgG as a reference (n = 3, mean ± S.D.). (**b**) The binding of HC-scTRAIL at three concentrations was analysed on Colo205 cells in absence or presence of a 200-fold molar excess of either cetuximab or TRAILR2-Fc for competition (n = 1, mean of triplicates ± S.D.). **(c)** The binding of hu225 IgG-scTRAIL fusion protein variants and hu225 IgG to EGFR-Fc was determined by ELISA (n = 3, mean ± S.D.). (**d**) The binding of scTRAIL fusion protein variants, hu225 IgG and Fc-scTRAIL-FAVSGAA to TRAILR1-Fc and TRAILR2-Fc was analysed by ELISA (n = 3, mean ± S.D.).

As shown by flow cytometry analysis on EGFR-positive human colon carcinoma cell lines Colo205 and HCT116, hu225 IgG and cetuximab bound with similar EC_50_ values to the target cells, demonstrating that the humanized IgG retained the EGFR binding activity (Supplemental Fig. S1). Next, we analysed whether fusion with scTRAIL has an influence on the binding properties of hu225 IgG to Colo205 and HCT116 (Fig. 2a, Table 2). Compared to hu225 IgG, all scTRAIL fusion protein variants bound with slightly higher EC_50_ values, but still within the sub-nanomolar range, suggesting that the affinity of scTRAIL to its cognate receptors has no major impact on the overall avidity of the proteins to EGFR^+^ cells at 4 °C. Interestingly, amongst the two hexavalent configurations, HC-scTRAIL exhibited EC_50_ values close to those of the hu225 IgG. Upon competition of binding of HC-scTRAIL to Colo205 cells using a 200-fold molar excess of cetuximab or TRAILR2-Fc, respectively, a contribution of TRAILR binding and EGFR binding could clearly be confirmed, with the latter having the dominant activity (Fig. 2b). In an EGFR ELISA, sub-nanomolar EC_50_ values were determined for binding of all IgG-scTRAIL fusion proteins to immobilized EGFR-Fc, although EC_50_ values were approximately 2- to 3-fold increased, compared to hu225 IgG (Fig. 2c, Table 2). Regarding TRAIL receptor binding, HC-scTRAIL bound with significantly lower EC_50_ values to TRAILR1 compared to LC-scTRAIL (P < 0.05), LC/HC-scTRAIL (P < 0.001) and Fc-scTRAIL-FAVSGAA (P < 0.05). Compared to TRAILR1, a stronger binding of the scTRAIL fusion proteins was observed for TRAILR2 with approximately 4- to 8-fold lower EC_50_ values (Fig. 2d, Table 2, Supplemental Fig. S2). Taken together, all three IgG-scTRAIL fusion proteins bound specifically and with high affinity to EGFR and both proapoptotic TRAIL receptors.

**Table 2:** EC_50_ values of protein binding to EGFR-positive tumour cells (flow cytometry), EGFR-Fc, TRAILR1-Fc and TRAILR2-Fc (ELISA), n = 3, mean ± SD.

Molecule	Colo205 (pM)	HCT116 (pM)	EGFR-Fc (pM)	TRAILR1-Fc (nM)*	TRAILR2-Fc (nM)*
hu225 IgG	60 ± 41	70 ± 21	156 ± 19	—	—
LC-scTRAIL	148 ± 83	181 ± 79	460 ± 27	8.0 ± 1.5	0.9 ± 0.1
HC-scTRAIL	81 ± 59	118 ± 33	343 ± 72	3.3 ± 0.6	0.8 ± 0.1
LC/HC-scTRAIL	146 ± 28	91 ± 72	315 ± 87	12.0 ± 1.6	3.4 ± 0.5
Fc-scTRAIL-FAVSGAA	n. d.	n. d.	—	7.3 ± 0.8	1.1 ± 0.1
hu225 IgG^#^	28 ± 6	8 ± 3	—	—	—
Cetuximab^#^	19 ± 8	9 ± 3	—	—	—

*Normalised to scTRAIL units; ^#^detection via anti-Fc antibody.

### Cell death induction by IgG-scTRAIL fusion proteins in tumour cells *in vitro*

**Figure 3:**
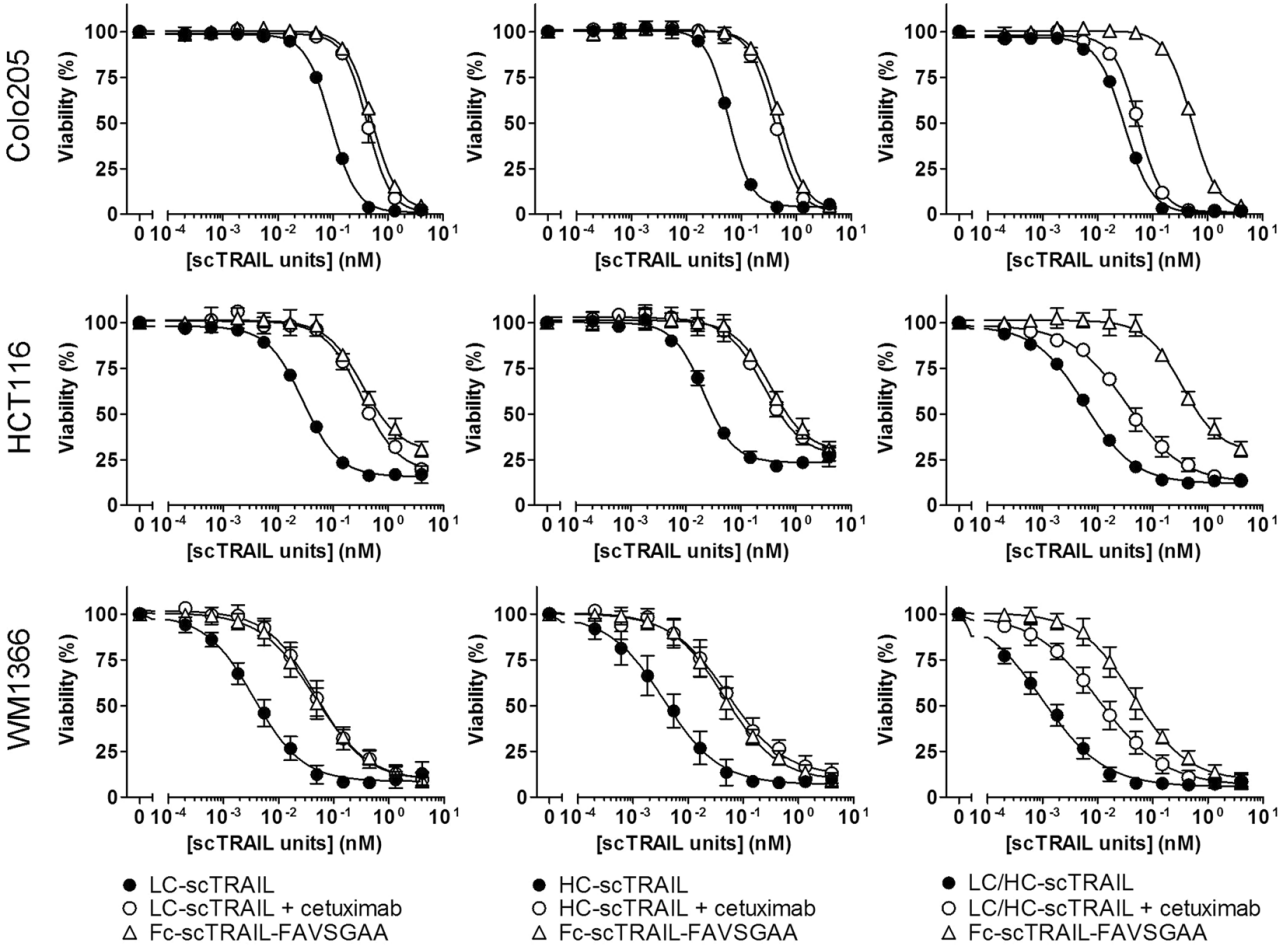
Cell death induction of IgG-scTRAIL proteins *in vitro*. The cell death inducing activity of hu225 IgG-scTRAIL fusion protein variants and Fc-scTRAIL-FAVSGAA in Colo205, HCT116 and WM1366 tumour cell lines was analysed *in vitro* by cell viability assays. Tumour cells were incubated with the proteins titrated in triplicates for 16 h, followed by crystal violet staining. For competition of EGFR targeting, the assay was performed likewise, but IgG-scTRAIL fusion proteins were co-incubated with 70 nM cetuximab, which was added 30 min prior to addition of the scTRAIL proteins (n = 3, mean ± S.D.).

Cell death-inducing activity of the EGFR-targeted IgG-scTRAIL fusion proteins in comparison to a non-targeted, hexavalent variant of the Fc-scTRAIL format (Fc-scTRAIL-FAVSGAA) was analysed *in vitro* using the EGFR^+^ tumour cell lines Colo205, HCT116 (both colon carcinoma) and WM1366 (malignant melanoma)^20^ (Fig. 3, Table 3). IgG-scTRAIL variants with a hexavalent TRAIL configuration (LC-scTRAIL and HC-scTRAIL) showed ~5- to ~22-fold lower EC_50_ values and therefore an increased bioactivity compared to Fc-scTRAIL-FAVSGAA, suggesting a clear benefit of EGFR targeting in terms of cell death induction by hexavalent TRAIL formats. Importantly, competition of EGFR binding by cetuximab abrogated the targeting effect completely, resulting in bioactivities at the level of Fc-scTRAIL-FAVSGAA. As expected from the increase of TRAIL valence, dodecavalent LC/HC-scTRAIL showed, when normalized to scTRAIL units, approximately 2- to 5-fold higher bioactivity compared to the two hexavalent formats. Interestingly, cetuximab only partially blocked the bioactivity of the LC/HC-scTRAIL on the tested tumour cell lines. Moreover, the co-incubation of the scTRAIL fusion proteins with the clinically established proteasome inhibitor bortezomib resulted in an up to 5-fold increase of bioactivity (Table 3, Supplemental Fig. S3).

**Table 3:** EC_50_ values (pM scTRAIL units) of protein bioactivity on EGFR-positive tumour cells (n = 3, mean ± S. D.).

	Colo205 w/o BZB	Colo205 + BZB	HCT116 w/o BZB	HCT116 + BZB	WM1366 w/o BZB	WM1366 + BZB
Fc-scTRAIL-FAVSGAA	508 ± 11	46.1 ± 0.4	713 ± 134	198 ± 19	57.6 ± 18.4	6.1 ± 2.1
HC-scTRAIL	58.5 ± 1.5	16.2 ± 1.4	31.5 ± 2.9	24.2 ± 1.5	4.7 ± 2.2	1.3 ± 0.6
HC-scTRAIL + Cetuximab	397 ± 7	48.1 ± 2.7	525 ± 62	244 ± 15	74.7 ± 28.6	13.6 ± 6.2
LC-scTRAIL	92.3 ± 2.4	21.5 ± 1.7	36.3 ± 1.4	29.4 ± 9.5	4.5 ± 1.1	1.6 ± 0.4
LC-scTRAIL + Cetuximab	422 ± 56	62.3 ± 2.4	467 ± 51	199 ± 67	62.9 ± 17.1	11.3 ± 2.7
LC/HC-scTRAIL	30.5 ± 0.9	5.6 ± 0.9	7.5 ± 0.4	5.7 ± 2.3	1.2 ± 0.4	0.3 ± 0.1
LC/HC-scTRAIL + Cetuximab	54.1 ± 6.1	8.8 ± 1.3	47.7 ± 13.0	23.5 ± 8.4	13.1 ± 5.0	2.7 ± 0.9

### *In vitro* stability and pharmacokinetics of HC-scTRAIL

**Figure 4:**
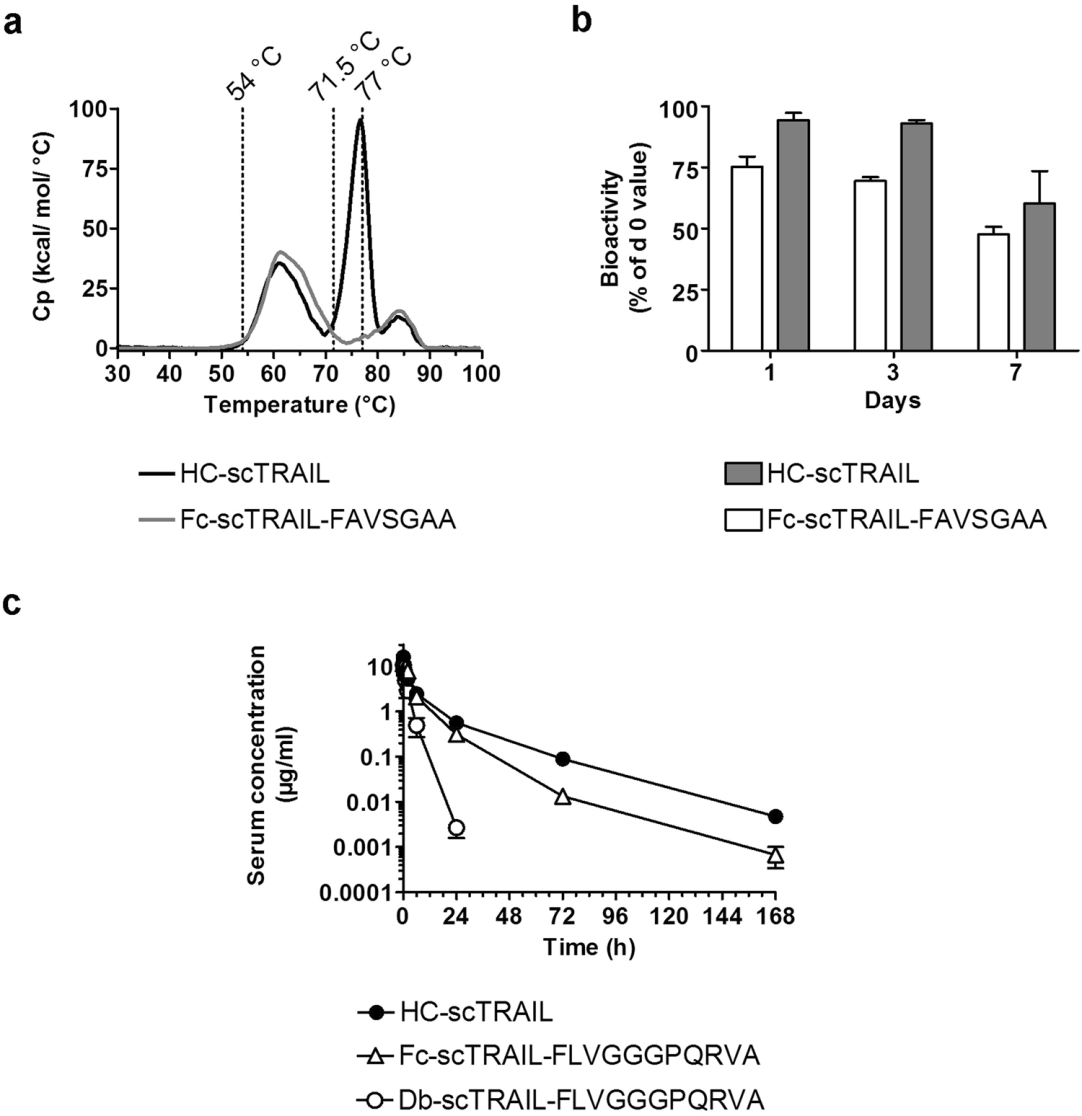
*In vitro* stability and pharmacokinetics of HC-scTRAIL. (**a**) The thermal stability of HC-scTRAIL and Fc-scTRAIL-FAVSGAA was analysed by differential scanning calorimetry. The onset temperatures of unfolding processes are indicated by dotted lines. **(b)** The bioactivities of HC-scTRAIL and Fc-scTRAIL-FAVSGAA were tested on Colo205 cells after incubating the proteins for different times at 37 °C in 50% human blood plasma (EC_50_ values normalized to non-incubated control, n = 1, mean of triplicates ± S.D.). **(c)** The serum concentrations after i.v. administration of 25 µg of HC-scTRAIL in CD-1 mice were analysed by ELISA. Values for Fc-scTRAIL-FLVGGGPQRVA and Db-scTRAIL-FLVGGGPQRVA are plotted for comparison and were adapted from ref.^17^ (n = 3, mean ± S.D.).

Due to its favourable characteristics in terms of expression titres, receptor binding, bioactivity and molecule size, we selected HC-scTRAIL for further studies and focused first on protein stability *in vitro*. Thermal stability of HC-scTRAIL protein moieties was analysed by differential scanning calorimetry revealing a first melting point at an onset temperature of 54 °C, a second at 71.5 °C, and a third at 77 °C (Fig. 4a). The first melting point can be attributed to the denaturation of the scTRAIL moiety as the most thermosensitive component of the molecule. This was confirmed by the thermogram of Fc-scTRAIL-FAVSGAA, which showed the same first melting point. This fusion protein showed a second melting point of 77 °C, which can be attributed to the Fc portion, corresponding to the third melting point of HC-scTRAIL. The second melting point of HC-scTRAIL is most likely caused by denaturation of the Fab domains.

The plasma stability of HC-scTRAIL and Fc-scTRAIL-FAVSGAA was assayed via cell death assay and ELISA (Fig. 4b, Supplemental Fig. S4). After one week of incubation at 37 °C in human blood plasma, the bioactivity of both proteins was at least 50%, which corresponded to ~70–90% intact protein as measured by ELISA.

Pharmacokinetic properties of IgG-scTRAIL (HC-scTRAIL) were determined in immunocompetent CD-1 mice receiving a single dose intravenous (i.v.) injection (Fig. 4c). The protein showed a terminal half-life t_1/2_β of 16.1 ± 2.6 h and an area under the curve (AUC) of 76 ± 11 (µg/ml)h.

### Anti-tumour activity of HC-scTRAIL in Colo205 mouse xenografts

**Figure 5:**
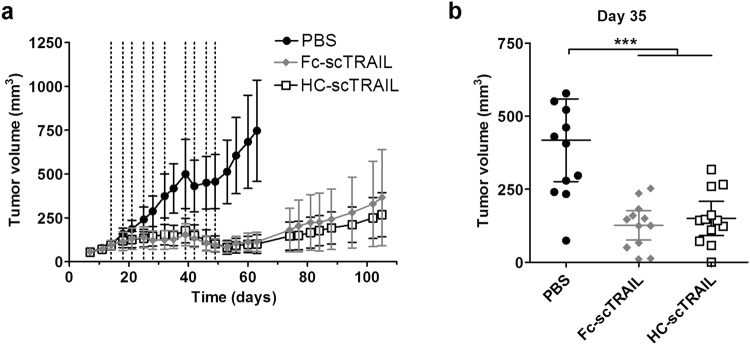
*In vivo* anti-tumour activity of HC-scTRAIL. **(a**) PBS or 0.3 nmol of either HC-scTRAIL or Fc-scTRAIL-FAVSGAA were administered i.v. to Colo205 bearing nu/nu mice twice a week for a total of six doses, starting from day 14. Administrations are indicated by dotted lines. At day 39, 100 µg Smac mimetic SM83 was administered i.p. as a co-treatment, together with a total of 4 i.v. doses of PBS or 0.3 nmol of the scTRAIL fusion proteins twice a week (n = 12 tumours, mean ± 95% C.I.). **(b)** Individual tumour volumes at day 35 (mean ± 95% C.I.).

Finally, we investigated the anti-tumour activity of HC-scTRAIL in the established nu/nu mouse xenograft model using subcutaneously implanted Colo205 cells (Fig. 5). When the tumours reached an average size of 100 mm^3^, six doses of HC-scTRAIL or Fc-scTRAIL-FAVSGAA as reference (0.3 nmol each) were administered i.v. twice a week (first cycle). In contrast to the progressively growing tumours of the PBS control group, both proteins inhibited the growth of the tumours significantly. In a second cycle, starting from day 39, animals were treated four times with a combination of scTRAIL fusion protein and intraperitoneally injected Smac mimetic SM83, which is known to synergistically enhance TRAIL-induced cell death^21^. Upon this combination treatment, we observed an additional ~50% reduction of the average tumour sizes until volumes of ~80 mm^3^ were reached at day 53. However, no difference regarding the monitored tumour volumes could be detected between the groups treated with EGFR-targeted HC-scTRAIL and non-targeted Fc-scTRAIL-FAVSGAA. Furthermore, no loss of body weight was observed, indicating that the administered doses of scTRAIL fusion proteins were well tolerated (Supplemental Fig. S5).

## Discussion

In this study we developed a novel, modular platform utilizing human IgG1 for generation of multivalent single-chain derivatives of the apoptosis-inducing TNF homology domain of TRAIL in combination with targeting of tumour-associated antigens, here demonstrated for EGFR. Fusing scTRAIL either to the C-terminus of the HC or LC, or to both chains of an IgG results in hexavalent or dodecavalent configurations of TRAIL. Our data demonstrate that EGFR binding in hexavalent LC-scTRAIL and HC-scTRAIL is functional and, compared to non-targeted hexavalent Fc-scTRAIL molecules, resulted in an enhanced bioactivity in three EGFR-expressing tested tumour cell lines *in vitro*. LC-scTRAIL and HC-scTRAIL fusion proteins exhibited similar EC_50_ values of cell death induction on these cell lines, indicating that fusion of the scTRAIL moieties to the C-terminus of both, the light and the heavy chain is suitable for generation of hexavalent IgG-scTRAIL fusion proteins. Furthermore, EGFR-dependent increase of apoptosis of tumour cells was independent of the sterical configuration of the molecule, i.e., the position of scTRAIL in the IgG molecule, as shown by competition with cetuximab. In addition to the hexavalent IgG-scTRAIL formats, we also showed that dodecavalent IgG-scTRAIL fusion proteins comprising 4 scTRAIL units can be produced as soluble proteins. Of note, this dodecavalent IgG-scTRAIL fusion protein, LC/HC-scTRAIL, exceeded the bioactivity of the hexavalent IgG-scTRAIL molecules. Most likely, this can be attributed to a fostered clustering of death receptors and consequently a more efficient DISC formation, especially of TRAILR2^22–24^.

Competition of LC/HC-scTRAIL with cetuximab only revealed a partial reduction of the bioactivity (e.g., approximately 2-fold on Colo205) compared to the hexavalent IgG-scTRAIL fusion proteins (~4-fold for LC-scTRAIL, ~7-fold for HC-scTRAIL on Colo205). In principle, cetuximab-competed LC/HC-scTRAIL was not expected to reach the EC_50_ values of the non-targeted Fc-scTRAIL fusion protein, because both molecules have different TRAIL valences. However, binding of the dodecavalent LC/HC-scTRAIL to EGFR and TRAIL receptors on EGFR^+^ tumour cells was shown to be similar compared to hexavalent formats, suggesting a rather stronger reduction of bioactivity than observed upon competition with cetuximab. In fact, this finding led us hypothesize that TRAIL valences higher than six may dominate over bivalent EGFR targeting. In this regard, an anti-proliferative effect of the cetuximab-derived hu225 IgG can be excluded for two reasons: (i) the setting and the short duration (16 h) of the cytotoxicity assay performed here were chosen to assess TRAIL-induced apoptosis and are rather not suitable to monitor tumour cell growth, and (ii) all analysed tumour cell lines carry activating mutations in MAPK pathway proteins downstream of EGFR (WM1366: NRAS^mut^, Colo205: BRAF^mut^, HCT116: KRAS^mut^), thereby abolishing anti-proliferative/pro-apoptotic effects of cetuximab due to constitutively active MAPK signalling which is independent of growth factor stimulation. Mechanistically, we therefore propose that the *in vitro* targeting effect of IgG-scTRAIL in the cell lines investigated here is predominantly mediated by a faster and more intense caspase activation, putatively in consequence of a rapid formation and/or stabilisation of TRAIL-TRAILR complexes, which is in line with observations from EGFR-targeted scFv-Fc-scTRAIL^17^. In principle, the hu225 V_H_ and V_L_ fragments used in IgG-scTRAIL have been shown to inhibit, as demonstrated for the Db-scTRAIL format, EGFR tyrosine autophosphorylation in a similar manner like cetuximab^13,21^, which can potentially result in additional growth inhibitory effects in EGFR/MAPK pathway wild-type tumour cells. However, the relevance of such effects for tumour therapy with IgG-scTRAIL is, in view of the much faster and robust apoptosis induction triggered by TRAIL, presumably insignificant.

For HC-scTRAIL we determined a terminal half-life in mice of approximately 16 h, which is slightly longer than the half-life of Fc-scTRAIL molecules but similar to a previously generated scFv-Fc-scTRAIL fusion protein also targeting the EGFR^17^. This supports recent findings demonstrating the importance of the Fc moiety for prolonged serum half-life, presumably by implementing FcRn-mediated recycling^17^. Interestingly, the HC-scTRAIL fusion protein, exhibiting a molecular mass and a Stokes radius exceeding that of the corresponding IgG, did not reach the long half-life of the IgG of approximately 9 days in mice^17^. Comparison of Fc-scTRAIL molecules comprising either wild-type or receptor-binding diminished scTRAIL units indicated a contribution of TRAIL-receptor-independent clearance effects to the half-life of scTRAIL fusion proteins in mice^17^. Irrespective of the mechanisms affecting *in vivo* half-life, the IgG-scTRAIL appears as a promising format for a highly active TRAIL therapeutic.

In a Colo205 xenograft tumour model, HC-scTRAIL demonstrated strong anti-tumour activity which was similar to that of a non-targeted Fc-scTRAIL. This finding is in accordance with recent results comparing an EGFR-targeting scFv-Fc-scTRAIL with Fc-scTRAIL in the same Colo205 model^17^. The apparent discrepancy to the *in vitro* data, demonstrating superior activity of tumour targeted *versus* non targeted hexavalent TRAIL molecules, but similar therapeutic potency in this particular *in vivo* tumour model is currently not fully understood, but important parameters begin to emerge. Thus both, antigen density and affinity of the targeting antibody in relation to the affinity of TRAIL to its cognate death receptors appear to be important^19^. Further studies using other *in vivo* tumour models are necessary to conclude on the benefit of targeting TRAIL to tumour cells for a maximum therapeutic output and safety. Especially for the dodecavalent IgG-scTRAIL format the role of tumour targeting for *in vivo* anti-tumour activity as well as safety appears of particular relevance, because excessive death receptor clustering may cause apoptosis in non-malignant, death receptor-positive tissues.

We also provide further data emphasizing the importance of combined therapy of IgG-scTRAIL with apoptosis sensitizers such as bortezomib and Smac mimetic SM83 to enhance cell death and sustained tumour eradication *in vivo*, respectively. Particularly regarding the recently reported pro-tumourigenic and pro-metastatic roles of the TRAIL/TRAILR system in TRAIL resistant tumour cells^25,26^, future work will show whether appropriate combinations of TRAIL formats like IgG-scTRAIL with sensitizers will result in complete and lasting tumour remissions.

In conclusion, we provide evidence for the therapeutic potential of a novel IgG-based format for the generation of multivalent scTRAIL fusion proteins. By realization of two bioactivity-enhancing principles in one molecule, i.e. hexa- or dodecavalent configuration of TRAIL and binding to EGFR-positive tumour cells, IgG-scTRAIL combines efficient cell death induction in tumour cells with targeted delivery. Though demonstrated here for EGFR targeting, the antigen versatility of the IgG-scTRAIL format facilitates the generation of tailor-made molecules to fight different cancer entities by addressing respective tumour-associated antigens on the cell surface. Moreover, considering the ambivalent role of TRAIL in certain scenarios, antigen targeting by TRAIL-based protein therapeutics could be a suitable strategy to ensure systemic safety and tumour cell-restricted bioactivity. Due to the heteromeric composition of the IgG building block, a future perspective is to combine scTRAIL moieties with a second, e.g. immune-stimulating, effector moiety within one covalently linked heterodimer, thus creating a multifunctional molecule for cancer treatment.

## Methods

### Cell lines and materials

HEK293, Colo205 and HCT116 cell lines were purchased from ATCC. The WM1366 cell line was generated at the WISTAR Institute. HEK293 were cultured in RPMI 1640 medium (Thermo Fisher) supplemented with 5% foetal bovine serum (FBS Premium or FBS Brazil One, PAN Biotech). Colo205, HCT116 and WM1366 were cultured in RPMI 1640 medium with 10% FBS and Pen-Strep (Thermo Fisher). During production of recombinant proteins, HEK293 cells were cultivated in Opti-MEM™ I (Thermo Fisher). HEK293-S cells were kindly provided by Dr. Verena Berger (Baliopharm GmbH, Jülich, Germany) and cultivated in Erlenmeyer shaker flasks in FreeStyle™ F17 Expression Medium (Thermo Fisher) supplemented with GlutaMAX™ (Thermo Fisher, 20 ml/L medium) and Kolliphor® P 188 (Sigma-Aldrich, 0.1% w/v). All cell lines were cultivated at 37 °C with 5% CO_2_. Bortezomib was from UBPBio. Polyethylenimine (PEI) was purchased from Polysciences GmbH and a 1 mg/ml stock solution was prepared as described^27^. Tryptone N1 was from Organotechnie. Human plasma was obtained from the blood bank of the Klinikum Stuttgart. Cetuximab was provided by Dr. Thomas Mürdter (IKP, Stuttgart) and SM83 was provided by BioNTech (Mainz, Germany).

### Generation of expression plasmids

The pEE6.4/ pEE14.4 double gene expression vector system was purchased from Lonza. In preparation for the scTRAIL fusion, we first inserted humanized variants of the C225 V_H_ and V_L_ domains (‘hu225’)^13^ into a human IgG1 backbone comprising C_H_1-C_H_2-C_H_3 with impaired ADCC/CDC functionality^28,29^ or C_Κ_, respectively. In opposition to the parental chimeric antibody cetuximab, the resulting molecule hu225 IgG is therefore entirely human. In detail, hu225 V_H_ and V_L_ were amplified by PCR from the previously published plasmid pCR3-Db-Glyco-scTRAIL-FAVSGAA^10^ and hu225 V_H_ was cloned via *Age*I/*Apa*I into the acceptor vector pEE6.4-huEmp1C_H_1-C_H_3, carrying already C_H_1-C_H_2-C_H_3, to yield pEE6.4-hu225-HC. Hu225 V_L_ was first subcloned into pAB1-huC_Κ_, carrying already C_Κ_, via *Age*I/*Rsr*II and then the entire light chain coding sequence was cloned via *Hin*dIII/*Eco*RI into pEE14.4 to yield pEE14.4-hu225-LC. For C-terminal fusion of the light chain with scTRAIL, scTRAIL-FAVSGAA was amplified by PCR from pCR3-Db-Glyco-scTRAIL-FAVSGAA and assembled with C_Κ_, amplified from pAB1-huC_Κ_, via overlapping DNA sequences, followed by a final PCR to amplify the entire assembly product. The product was cut *Rsr*II/*Mfe*I and cloned into *Rsr*II/*Eco*RI cut pEE14.4-hu225-LC, yielding pEE14.4-hu225-LC-scTRAIL. For heavy chain fusion with scTRAIL, the scTRAIL-FAVSGAA PCR product was assembled with a DNA fragment comprising CH1-CH2-CH3, amplified from pEE6.4-huEmp1C_H_1-C_H_3, via overlapping DNA sequences, followed by a final PCR to amplify the entire assembly product. The product was 5′ phosphorylated with T4 polynucleotide kinase, cut with *Apa*I and cloned into *Eco*RI cut, mung bean nuclease digested, *Apa*I cut pEE6.4-hu225-HC to yield pEE6.4-hu225-HC-scTRAIL. Finally, double gene fusion vectors from the combinations pEE6.4-hu225-HC/pEE14.4-hu225-LC (for hu225 IgG), pEE6.4-hu225-HC/pEE14.4-hu225-LC-scTRAIL (for LC-scTRAIL), pEE6.4-hu225-HC-scTRAIL/pEE14.4-hu225-LC (for HC-scTRAIL) and pEE6.4-hu225-HC-scTRAIL/pEE14.4-hu225-LC-scTRAIL (for LC/HC-scTRAIL) were obtained via *Bam*HI/*Not*I cloning. The Fc-scTRAIL-FAVSGAA expression construct was generated by PCR amplification of FLAG-Fc(Q) from pSecTag-FLAG Fc(Q) scTRAIL 118 G^17^. The coding sequence of the ‘glyco’ linker was added 3′ via PCR, followed by assembly with a scTRAIL-FAVSGAA DNA fragment via overlapping sequences and replacement of the coding sequence in pSecTag-FLAG Fc(Q) scTRAIL 118G by *Age*I/*Xba*I cloning.

### Production and purification of recombinant proteins

In preparation for transient production of scTRAIL proteins, HEK293 cells were grown to 50–75% confluence in T175 flasks and on day of transfection, the culture medium was replaced by fresh medium. 50 µg plasmid DNA and 150 µg PEI were each mixed in 2.5 ml Opti-MEM™ I, combined and vortexed for 3 × 1 s, incubated for 20 min at room temperature and given to the flask, followed by careful swinging. 16 h after transfection, the medium was replaced by Opti-MEM™ I medium + 50 µM ZnCl_2_ and cells were cultivated for 3 days. For extended protein productions, genetically engineered HEK293-S suspension cells (M. Siegemund, unpublished data) were transiently transfected. The cells were grown to 1.5–2.0 × 10^6^ cells/ml and transfection was done by diluting 1 µg plasmid DNA/ ml culture and 3 µg PEI/ml culture each in fresh culture medium (1/20 of culture volume). Both solutions were combined, treated as described above and given to the culture flask, followed by immediate shaking. 24 hours after, 0.5% tryptone N1 and 10 µM ZnCl_2_ were added and cells were grown for another 5 days. All cultures were harvested by centrifugation at 2,000 × *g* for 30 min at 4 °C and proteins were purified from supernatants by anti-FLAG affinity chromatography as described^30^. To this, supernatants were incubated with anti-FLAG® M2 Affinity Gel (Sigma-Aldrich, 3 ml bead volume/500 ml supernatant) for at least 2 h at 4 °C on a roller mixer. Beads were collected in an empty column and washed with 5 CV 1 × TBS. Bound proteins were eluted with 7 CV 100 µg/ml DYKDDDDK peptide/1 × TBS (peptides&elephants). After dialysis in 1 × PBS, eluates were concentrated with Vivaspin® 20 devices, 50 kDa MWCO (Sartorius). In combination with production in genetically engineered HEK293-S cells, HC-scTRAIL and Fc-scTRAIL-FAVSGAA were further purified by preparative gel filtration on a Superdex® 200 10/300 GL column (GE Healthcare). Protein concentrations were determined spectrophotometrically using the calculated extinction coefficients. Aliquots were stored at −80 °C. TRAILR1-Fc, TRAILR2-Fc and EGFR-Fc were produced and purified as previously described^15^.

### Biochemical and biophysical protein analysis

Purified proteins were analysed by SDS-PAGE under non-reducing and reducing conditions, followed by staining with InstantBlue™ (Expedeon). For SEC, a TSKgel® SuperSW mAb HR, 7.8 × 300 mm column (Tosoh) was equilibrated in 0.1 M Na_2_HPO_4_/NaH_2_PO_4_, 0.1 M Na_2_SO_4_, pH 6.7 and proteins were eluted at a flow rate of 0.5 ml/min. The globular proteins thyroglobulin (669 kDa, R_s_ 8.5 nm), apoferritin (443 kDa, R_s_ 6.1 nm), β-amylase (200 kDa, R_s_ 5.4 nm), BSA (66 kDa, R_s_ 3.55 nm) and carbonic anhydrase (29 kDa, R_s_ 2.35 nm) were used as references. Denaturation temperatures of proteins were measured by differential scanning calorimetry using a MicroCal DSC instrument (Malvern).

### ELISA

96-well plates were coated with TRAILR1-Fc, TRAILR2-Fc (200 ng/well in 0.1 M sodium carbonate buffer, pH 9.5) or EGFR-Fc (300 ng/well in PBS) overnight at 4 °C and then blocked with 3% (w/v) skim milk powder in PBS (MPBS). The protein samples were diluted in MPBS, titrated 1:3 in duplicates starting from 150 nM (TRAILR ELISA) or 50 nM (EGFR ELISA) and incubated on the receptor-Fc-coated plates for 2 h at room temperature on a shaker. Relevant for TRAILR binding, molar concentrations of the samples were normalized to one scTRAIL unit. Bound protein was for TRAILR ELISA detected by 1:15,000 in MPBS diluted monoclonal anti-FLAG® M2-peroxidase (HRP) antibody (Sigma-Aldrich) or for EGFR ELISA by 1:10,000 in MPBS diluted anti-human IgG (Fab specific)-peroxidase antibody (Sigma-Aldrich) in combination with 3,3′,5,5′-tetramethylbenzidine (TMB) as substrate (0.1 mg/ml TMB, 100 mM sodium acetate buffer, pH 6.0, 0.006% H_2_O_2_). Reactions were stopped with 50 µl of 2 M sulfuric acid and absorbance was measured at 450 nm. Data were fitted with Prism (GraphPad Software) in order to calculate EC_50_ values ± SD from three independent experiments. EC_50_ values of binding were analysed by unpaired t-test or one-way ANOVA and Tukey post hoc tests for statistical evidence.

### Flow cytometry

Cells were trypsinised and then washed once and re-suspended in FACS buffer (PBS, 2% FBS, 0.05% NaN_3_). 1.5 × 10^5^ cells were incubated for 2 h on ice in presence of serially diluted proteins in FACS buffer starting from 50 nM or without protein (control). For competition experiments, cells were incubated with a 200-fold molar excess of cetuximab or TRAILR2-Fc for 30 min on ice before the proteins were added. The cells were washed two times with ice-cold FACS buffer, followed by incubation of protein samples and controls with 1:100 in FACS buffer diluted anti-DYKDDDDK-PE labelled antibody (Miltenyi Biotec) on ice for 1 h. Cetuximab and hu225 IgG binding was detected with 1:500 in FACS buffer diluted anti-human IgG (γ chain specific)-R-phycoerythrin antibody (Sigma-Aldrich). After two washing steps, cells were analysed on a MACSQuant® Analyzer 10 equipped with a 585/40 nm filter or a MACSQuant® VYB with 586/15 nm filter (Miltenyi Biotec). Data were fitted with Prism from three independent binding curves.

### Cell death assays

Colo205 (4 × 10^4^/well), HCT116 (1.5 × 10^4^/well) and WM1366 (2 × 10^4^/well) cells were seeded in 100 µl culture medium in 96-well plates and grown for 24 h, followed by treatment with serial dilutions (1:3) of proteins in triplicates. Molar concentrations were normalized to one scTRAIL unit. For positive control, cells were lysed with 0.5% Triton™ X-100. Cell death assays were performed without sensitization of carcinoma cells or in the presence of the proteasome inhibitor bortezomib (250 ng/ml for Colo205, 5 ng/ml for HCT116, 250 ng/ml for WM1366; UBPBio), which was added 30 min prior incubation with the proteins. After 16 h of incubation, cell viability was determined by crystal violet staining. Alternatively, to demonstrate EGFR-dependent enhancement of cell death, cells were pre-incubated for 30 min with 70 nM of cetuximab for competition. Data were analysed with Prism and EC_50_ values ± SD were calculated, based on three independent experiments.

### Plasma stability assay

Proteins were incubated at a concentration of 0.5 µM in 50% human blood plasma. The bioactivity was analysed by cell death assay on Colo205 cells, as described before, without incubation of the samples at 37 °C (control) or after 1, 3 and 7 days at 37 °C, respectively. EC_50_ values ± SD were calculated with Prism from triplicates and normalized to the control.

### Pharmacokinetic studies

Animal care and all experiments performed were in accordance with federal and European guidelines and have been approved by university (animal welfare officer) and state authorities (Regierungspräsidium Stuttgart). An i.v. injection of 25 µg of HC-scTRAIL was administered to female CD-1 mice (Charles River, 8 weeks old, 3 animals). Blood samples (50 µl) were taken at 0.05, 0.5, 1, 2, 6, 24, 72 and 168 hours and incubated on ice for 30 minutes. Clotted blood was centrifuged at 13,000 × *g* for 30 minutes, 4 °C and serum samples were stored at −20 °C. The serum concentration of HC-scTRAIL was analysed with BD OptEIA™ Human TRAIL ELISA Set (BD) according to the manufacturer’s instructions. The first value (0.05 h) was set to 100%. The initial (t_½_α) and terminal (t_½_β) half-lives and the bioavailability (area under the curve, AUC) were calculated with MS Excel.

### Xenograft mouse tumour model

Female NMRI nu/nu mice (Charles River, 12 weeks old) received s.c. injections of 3×10^6^ Colo205 cells in 100 µl PBS at the left and right dorsal sides. Treatment started 14 days after tumour cell inoculation when tumours reached an average volume of 100 mm^3^. 0.3 nmol HC-scTRAIL or Fc-scTRAIL-FAVSGAA were administered twice a week in 100 µl PBS for a total of six doses. The control group received six doses of 100 µl PBS. In a second round of treatment, 100 µg Smac mimetic SM83 was co-administered i.p., together with a total of 4 i.v. doses of PBS or 0.3 nmol of the proteins twice a week. Tumour volume was monitored as described^9^. One-way ANOVA and Tukey post hoc tests were performed for statistical analysis.

### Data availability statement

All data generated or analysed during this study are included in this published article (and its Supplementary Information files).

## Electronic supplementary material

Supplementary Figures S1-S5
