# Supplementary material for: IgG-single-chain TRAIL fusion proteins for tumour therapy
Source: Sci Rep. 2018 May 17;8:7808. doi: 10.1038/s41598-018-24450-8 (PMC5958125; doi:10.1038/s41598-018-24450-8)
Supplement: Supplementary file 1 — Supplementary Figures S1-S5 [file 41598_2018_24450_MOESM1_ESM.docx]

**Supplementary Information**

**IgG-single-chain TRAIL fusion proteins for tumour therapy**

Martin Siegemund, Felix Schneider, Meike Hutt, Oliver Seifert, Ines Müller, Dagmar Kulms, Klaus Pfizenmaier and Roland E. Kontermann

**Supplemental Figure S1: Comparison of hu225 IgG and cetuximab.** The binding of fully human/humanized hu225 IgG and parental chimeric cetuximab to EGFR-positive colon carcinoma cell lines Colo205 and HCT116 was tested by flow cytometry (n = 3, mean ± S. D.).

**Supplemental Figure S2: Biochemical analysis of Fc-scTRAIL-FAVSGAA. (a)** Non-reducing and reducing SDS-PAGE of Fc-scTRAIL-FAVSGAA. **(b)** Size exclusion chromatography of Fc-scTRAIL-FAVSGAA.

**Supplemental Figure S3: Cell death induction of IgG-scTRAIL proteins *in vitro* in presence of bortezomib.** The cell death inducing activity of hu225 IgG-scTRAIL fusion protein variants and Fc-scTRAIL-FAVSGAA in Colo205, HCT116 and WM1366 tumour cell lines was analysed *in vitro* by cell viability assays in presence of the proteasome inhibitor bortezomib (BZB, 250 ng/ml for Colo205, 5 ng/ml for HCT116 and 250 ng/ml for WM1366) as apoptosis sensitiser. Tumour cells were incubated with the proteins titrated in triplicates for 16 h, followed by crystal violet staining. For competition of EGFR targeting, the assay was performed likewise, but IgG scTRAIL fusion proteins were co-incubated with 70 nM cetuximab, which was added together with the BZB 30 min prior to addition of the scTRAIL proteins (n = 3, mean ± S.D.).

**Supplemental Figure S4: Plasma stability of proteins.** The plasma stability of HC-scTRAIL and Fc-scTRAIL-FAVSGAA was analysed by ELISA. Proteins were incubated for different times at 37 °C in 50 % human blood plasma, bound to TRAILR2-Fc and detected via anti-FLAG HRP-conjugated antibody. EC_50_ values of binding were normalized to that of a control which was not incubated at 37 °C (n = 1, mean of duplicates).

**Supplemental Figure S5: Body weight.** The body weight of mice treated with the scTRAIL fusion proteins or PBS was monitored during the course of the experiment. Administrations are indicated by dotted lines.

**Supplemental Figure S1:**

**
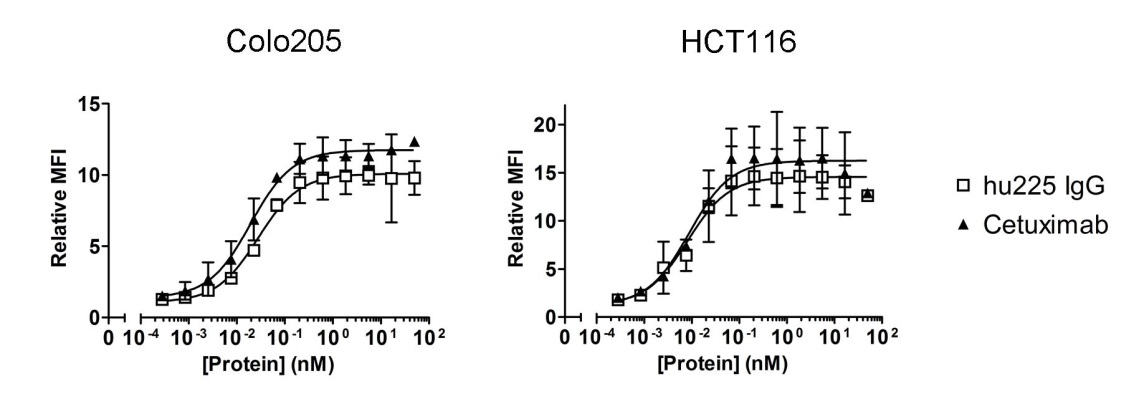
**

**Supplemental Figure S2:**


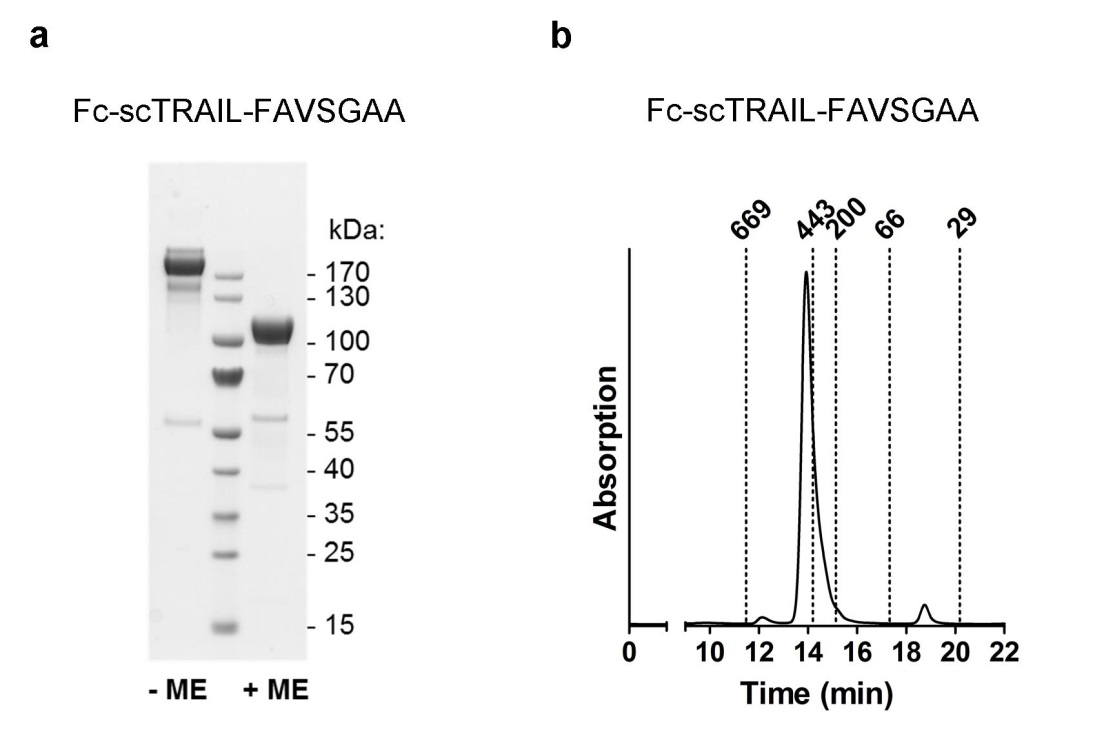


**Supplemental Figure S3:**

**
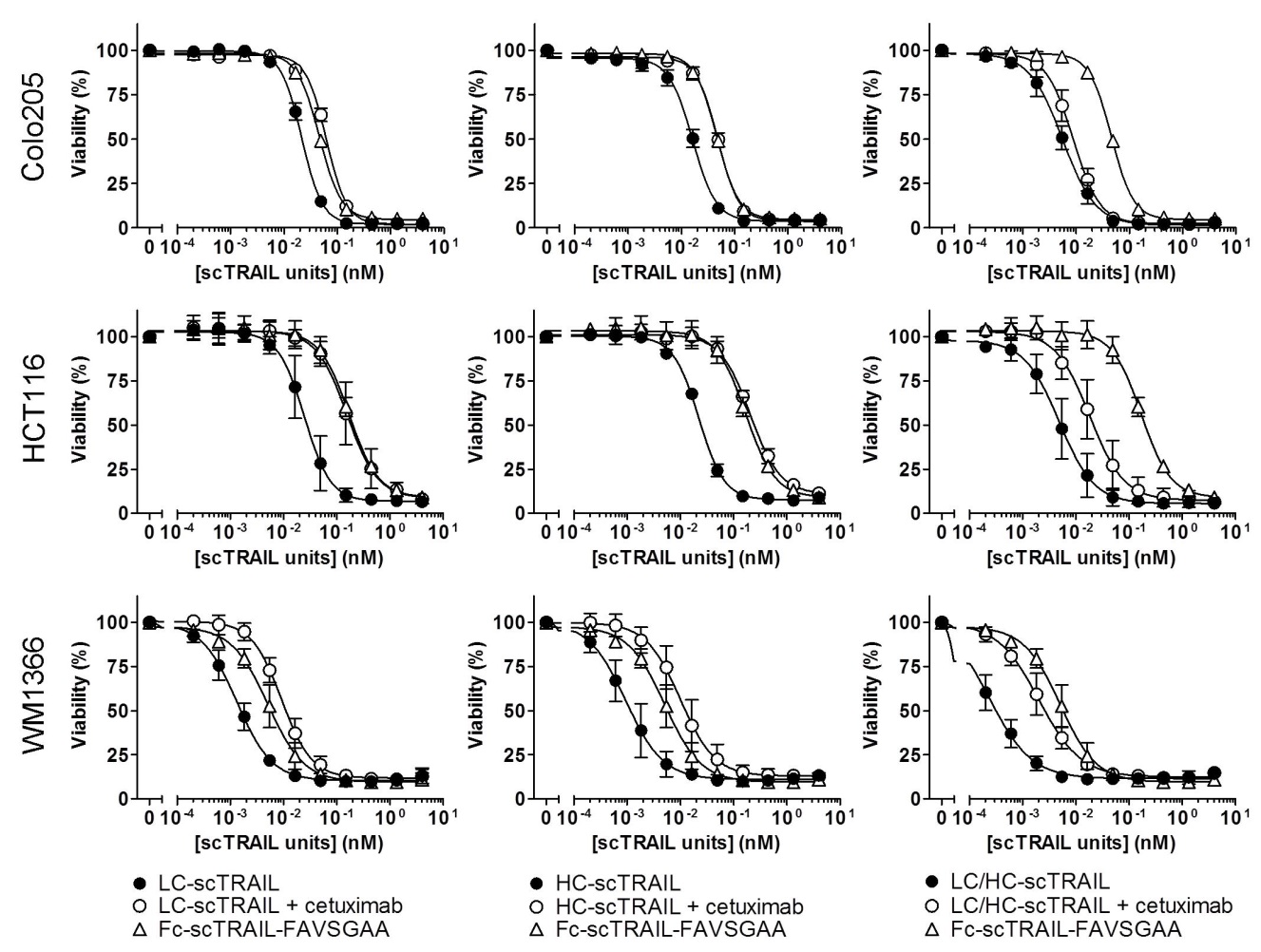
**

**Supplemental Figure S4:**

**
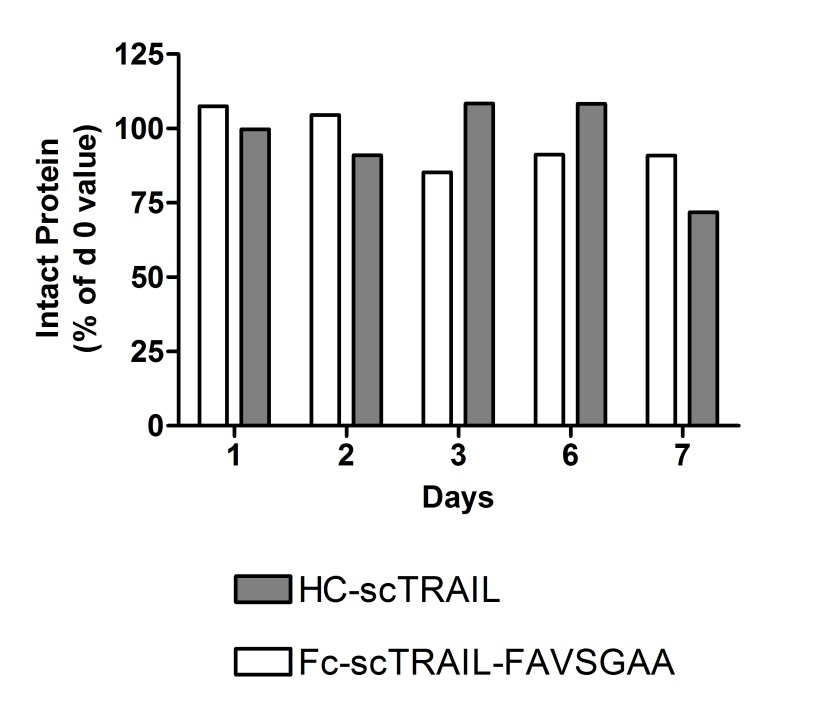
**

**Supplemental Figure S5:**

**
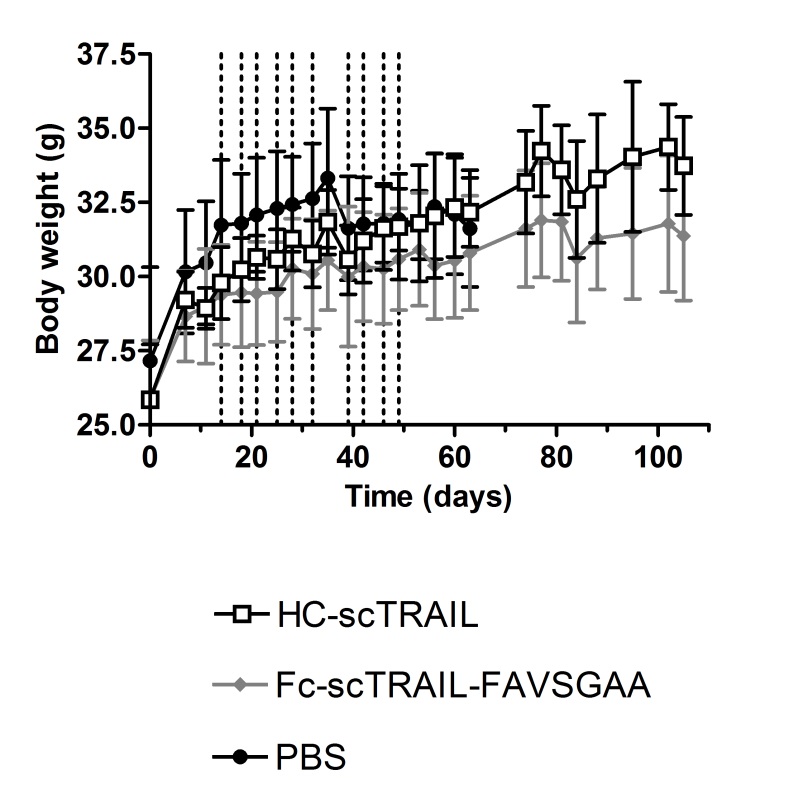
**
